# Associations of physical activity and sedentary time with health-related quality of life in patients with localized renal cell cancer: a cross-sectional analysis within the ReLife study

**DOI:** 10.1007/s00520-024-08969-3

**Published:** 2024-11-18

**Authors:** Alina Vrieling, Jake S. F. Maurits, Job Gerritsen, Laurien M. Buffart, Katja K. H. Aben, J. P. Michiel Sedelaar, Esmée A. Bakker, Lambertus A. L. M. Kiemeney

**Affiliations:** 1 IQ Health Science Department, Radboud University Medical Center, Kapittelweg 54, 6525EP Nijmegen, The Netherlands; 2 Department of Medical BioSciences, Radboud University Medical Center, Nijmegen, The Netherlands; 3 Netherlands Comprehensive Cancer Organisation, Department of Research and Development, Utrecht, The Netherlands; 4 Department of Urology, Radboud University Medical Center, Nijmegen, The Netherlands

**Keywords:** Physical activity, Sedentary time, Self-reported, Accelerometry, Quality of life, Renal cell cancer

## Abstract

**Purpose:**

This study examined the associations of device-measured moderate-to-vigorous physical activity (MVPA) and sedentary time as well as self-reported MVPA with health-related quality of life (HRQoL) in patients with localized renal cell cancer (RCC) in the recovery phase after surgery.

**Methods:**

At 3 months post-surgery, 341 patients with stage I-III RCC participating in the ReLife study wore an ActivPAL3 device to determine MVPA and sedentary time. The SQUASH questionnaire was used for assessing self-reported MVPA, and the EORTC QLQ-C30 for assessing HRQoL (range 0–100). Multivariable linear regression models were used to examine the cross-sectional associations of MVPA and sedentary time with HRQoL.

**Results:**

The highest (≥ 6.7 h/week) versus lowest (≤ 2.7 h/week) quartile of MVPA was associated with a better global health status (*β*, 10.2; 95% CI, 5.1, 15.3), summary score (*β*, 4.6; 95% CI, 1.1, 8.1), physical (*β*, 7.7; 95% CI, 3.8, 11.6), role (*β*, 12.4; 95% CI, 4.7, 20.2), and social functioning (*β*, 7.3; 95% CI, 0.2, 14.4), and lower fatigue (*β*, − 11.2; 95% CI, − 18.1, − 4.2). Results for self-reported MVPA were in the same direction but weaker. The lowest (≤ 8.8 h/day) versus highest (≥ 11.5 h/day) quartile of sedentary time was associated with better physical functioning (*β*, 4.6; 95% CI, 0.8, 8.5).

**Conclusions:**

In patients with localized RCC, higher MVPA 3 months post-surgery was associated with better HRQoL outcomes including less fatigue whereas lower sedentary time was only associated with better physical functioning. This information can contribute to the development of physical activity guidelines and interventions to improve HRQoL.

## Introduction

Kidney cancer is the 14th most common cancer worldwide and about 90% of patients with kidney cancer are diagnosed with renal cell cancer (RCC) [1]. The majority of patients present with localized RCC, which is primarily treated with radical or partial nephrectomy [2]. In contrast to most of the other cancer types, radiotherapy, chemotherapy, and immunotherapy are usually not given since effectiveness has not been proven [2]. Overall health status including pain, gastrointestinal function, cognition, and activity as well as physical quality of life are reduced at 2 weeks after RCC surgery compared to before surgery [3]. At 12 weeks after RCC surgery, about 70–80% of patients are likely to have fully recovered [3]. However, patients may still suffer from side effects such as irritability, pain, worry, sleep disturbance, and fatigue [4]. These side effects may have an important impact on patients’ health-related quality of life (HRQoL) [5]. Thus, it is critical to investigate how HRQoL could be improved.

There is increasing evidence in cancer survivors that higher physical activity is associated with better HRQoL [6] while associations for sedentary time are inconsistent [7]. According to the World Cancer Research Fund/American Institute for Cancer Research (WCRF/AICR), cancer survivors should engage in at least 150 min/week of moderate-to-vigorous physical activity (MVPA) and minimize their sedentary time [8], similar to the recommendations of the World Health Organization [9]. To date, only one cross-sectional study investigated the association of physical activity with HRQoL among patients with RCC [10]. Significant dose–response associations of MVPA with HRQoL were observed from completely sedentary to 150–290 min/week of MVPA, with no further improvements in HRQoL for ≥ 300 min/week of MVPA. However, physical activity and HRQoL were assessed on average 6 years after diagnosis, which does not reflect the recovery phase after surgery. Also, physical activity was self-reported instead of being objectively measured via an accelerometer. The correlation of self-reported with device-measured MVPA [11] and sedentary time [12] has been reported to be low, and self-reported physical activity measures often result in overestimation of MVPA [13] and underestimation of sedentary time [14]. Questionnaires and accelerometers measure different aspects of physical activity and sedentary time [11–14]. Device-measured physical activity may provide a better understanding of how MVPA and sedentary time are related to HRQoL in patients with localized RCC. This information is important since it may contribute to informing future physical activity guidelines and interventions to improve HRQoL in the first months after surgery.

The main aim of this study was to investigate the association of device-measured MVPA and sedentary time with HRQoL in patients with localized RCC at 3 months after surgery. In addition, we assessed the correlation and bias between self-reported and device-measured MVPA, as well as the association of self-reported MVPA with HRQoL, We hypothesized that higher MVPA and lower sedentary time are associated with better HRQoL. We further postulated that the correlation between device-measured and self-reported MVPA is low, but that associations for device-measured and self-reported MVPA with HRQoL are similar.

## Methods

### Study design and population

The current cross-sectional analysis was performed using data from the Dutch multicenter cohort study ReLife (Renal Cell Cancer: Lifestyle, Prognosis, and Quality of Life) which has been described in detail elsewhere [15]. ReLife includes patients between the ages of 18 and 75 years who were newly diagnosed with a pathology-confirmed primary stage I, II, or III RCC tumor without lymph node or distant metastases and treated with surgery or ablation. Patients were recruited from January 2018 to June 2021 and requested to wear an accelerometer and to complete questionnaires at 3 months, 1 year, and 2 years after surgery. For this analysis, only data collected at 3 months after surgery were used, reflecting the recovery phase after surgery. A total of 882 patients were invited, of whom 46 appeared to be ineligible (reasons: deceased (*n* = 1), invitation letter could not be delivered (*n* = 3), did not fulfill the inclusion criteria due to stage IV disease (*n* = 16), previous tumor within past 5 years (*n* = 10), no RCC (*n* = 6), no surgery (*n* = 7), other reasons (*n* = 3)). In total, 368 of the 836 eligible patients agreed to participate (response rate 44%) [15]. Relative to non-responders, participants were more likely to be female (23% vs. 30%) but were similar regarding age, tumor stage, tumor grade, tumor morphology, and type of treatment [15]. After the exclusion of participants who did not wear the accelerometer at 3 months after surgery (*n* = 22), did not wear it for at least 3 consecutive days (*n* = 2), or did not complete the first questionnaire (*n* = 3), 341 participants were included in the current analysis. The study was in line with the principles of the Declaration of Helsinki. The study was approved by the Committee for Human Research region Arnhem-Nijmegen (CMO 2016–3078). Patients who agreed to participate provided written informed consent.

### Physical activity and sedentary behavior

Objective data on MVPA and sedentary time were collected using the validated ActivPAL3 accelerometer (PAL Technologies Ltd., Glasgow, UK) [16]. Patients attached the ActivPAL3 with a waterproof sleeve to the center of the right thigh 10 cm above the knee using Tegaderm film (Tegaderm, 3 M Medical) according to written instructions. Participants were instructed to wear the activPAL3 for seven consecutive days, 24 h per day. The raw acceleration data from the accelerometers was transferred to the associated activPAL software version 8. Subsequently, the data was transformed into analyzable data using a customized algorithm written in MATLAB R2018b (MathWorks. Natick, MA, USA) [17]. The time spent in MVPA was estimated by summing the time spent in cadences (i.e., steps per minute) of ≥ 100 and expressed as h/week [18]. Additionally, sedentary time was estimated by summing the time spent in sitting, lying, and reclining posture, excluding algorithm-based sleeping time, and expressed as hours/day.

Self-reported data on MVPA was derived from the validated short questionnaire to assess health-enhancing physical activity (SQUASH) [19]. The reference period was a normal week during the past 3 months. Participants reported the average time spent on commuting, work, household, and leisure-time activities (walking, cycling, gardening, odd jobs, and up to four sports). The weekly time spent on MVPA was calculated by summing all activities with a metabolic equivalent of task value ≥ 3, based on the Ainsworth compendium of physical activities [20].

### Covariates

Socio-demographic and lifestyle factors were collected via paper-and-pencil- or web-based questionnaires, as previously described [15]. These factors included among others age, biological sex, educational level (categorized as low, intermediate, or high), body weight, height, and cigarette smoking status (never, former, current). Body mass index (BMI in kg/m^2^) was calculated by dividing self-reported body weight by height squared and categorized according to the WHO classification [21] into underweight (BMI < 18.50), normal weight (BMI 18.50–24.99), overweight (BMI 25.00–29.99), and obesity (BMI ≥ 30.00). Information on 14 comorbidities was collected using an adapted version of the comorbidity questionnaire [22], and categorized as having 0, 1, or ≥ 2 comorbidities. Information on having any difficulties with walking in the previous week was also obtained by questionnaire and defined as mobility limitation (no, yes). Clinical factors were retrieved from the medical records by data managers of the Netherlands Comprehensive Cancer Organisation (IKNL) and included clinical and post-surgical TNM stage, tumor grade, type of surgery or ablation, and postoperative grade ≥ 2 complications graded using the Clavien-Dindo classification [23].

### Health-related quality of life

Data on HRQoL was acquired using the validated cancer-specific European Organization for the Research and Treatment of Cancer Quality of Life Questionnaire-Core 30 (EORTC QLQ-C30, version 3.0) [24]. The EORTC QLQ-C30 includes five functioning scales (i.e., physical, role, emotional, cognitive, social), three symptom scales (i.e., fatigue, nausea/vomiting, and pain), six single items (i.e., dyspnea, insomnia, loss of appetite, constipation, diarrhea, and financial difficulties), all scored from 1 (not at all) to 4 (very much), and a global health status scale ranging from 1 (very poor) to 7 (excellent). A summary score was calculated using all scales except global quality of life and financial difficulties [25]. All scales were linearly transformed to 0–100 scales. Higher scores indicated better HRQoL, except for the symptom scales and single items where higher scores implied worse HRQoL. The current analysis was focused on global health status, the five functioning scales, the summary score, and fatigue, since these were considered most likely to be influenced by MVPA and sedentary time [6].

### Statistical analysis

Descriptive characteristics were calculated for the total study population and stratified by device-measured MVPA and sedentary time quartiles, respectively. Spearman’s correlation coefficient was calculated to assess convergent validity, i.e., the degree to which device-measured and self-reported MVPA are related. A Bland–Altman plot was used to evaluate bias, with 95% limits of agreement for the total error between the two methods. Agreement on classifying participants as adhering (≥ 150 min/week) and non-adhering (< 150 min MVPA/week) to the WCRF/AICR physical activity guidelines [8] was assessed using Cohen’s Kappa statistic.

To examine the association of device-measured and self-reported MVPA and device-measured sedentary time with HRQoL, MVPA and sedentary time were analyzed as quartiles and as continuous variables. In addition, adherence to the WCRF/AICR physical activity guidelines was examined. Multivariable linear regression models were constructed to estimate regression coefficients (*β*) and corresponding 95% confidence intervals (CIs). The least beneficial group (i.e., low MVPA or high sedentary time) was used as the reference. All models were adjusted for age, sex, BMI, comorbidities, tumor stage, smoking status, education level, and mobility limitation. These variables were selected a priori based on literature [7, 10, 26]. Furthermore, models for device-measured MVPA and sedentary time were mutually adjusted. Multicollinearity was investigated but none was found. The clinical relevance of the differences in HRQoL scales was assessed using the guidelines for cross-sectional differences, published by Cocks et al. [27].

R version 4.1.3 (R Foundation for Statistical Computing, Vienna, Austria) was used for all calculations and analyses. *P*-values < 0.05 were considered statistically significant.

## Results

### Participant characteristics

The study population predominantly consisted of males (71.3%) and had a mean age of 62.8 (SD 8.9) years (Table 1). The majority of participants were overweight or obese (69.3%), had stage I RCC (64.6%), were treated with radical nephrectomy (57.8%), and reported at least two comorbidities (61.7%). Participants with the lowest (versus highest) device-measured MVPA and participants with the highest (versus lowest) sedentary time more frequently reported to be male, to have a higher BMI, and to be current smokers. Also, they are more frequently reported to have two or more comorbidities, a grade 2 or higher complication, and a mobility limitation.

**Table 1:** Descriptive characteristics for the total study population of patients with localized RCC and by quartiles of device-measured moderate-to-vigorous activity (MVPA) and sedentary time

		MVPA quartiles (h/week)				Sedentary time quartiles (h/day)			
	Total	0–2.7	2.8–4.2	4.2–6.7	≥ 6.7	≥ 11.4	10.2–11.4	8.8–10.2	0–8.8
Number of participants, *n*	341	85	85	85	86	86	85	85	85
Age (years), mean (SD)	62.8 (8.9)	66.5 (6.5)	61.4 (9.3)	62.0 (10.6)	61.4 (7.7)	62.9 (8.7)	64.7 (8.4)	61.4 (9.0)	62.2 (9.1)
Gender (male), *n* (%)	243 (71.3%)	65 (76.5%)	58 (68.2%)	60 (70.6%)	60 (69.8%)	68 (79.1%)	66 (77.6%)	61 (71.8%)	48 (56.5%)
Body mass index (kg/m^2^), mean (SD)	27.5 (4.6)	28.9 (5.2)	27.8 (4.4)	27.3 (4.7)	25.9 (3.5)	28.6 (4.8)	27.8 (4.5)	27.3 (4.4)	26.2 (4.4)
Body mass index categories (kg/m^2^), *n* (%)									
< 25^a^	104 (30.5%)	14 (16.5%)	27 (31.8%)	27 (31.8%)	36 (41.9%)	16 (18.6%)	25 (29.4%)	28 (32.9%)	35 (41.2%)
25–30	156 (45.7%)	39 (45.9%)	38 (44.7%)	42 (49.4%)	37 (43.0%)	43 (50.0%)	36 (42.4%)	42 (49.4%)	35 (41.2%)
≥ 30	81 (23.8%)	32 (37.6%)	20 (23.5%)	16 (18.8%)	13 (15.1%)	27 (31.4%)	24 (28.2%)	15 (17.6%)	15 (17.6%)
Education, *n* (%)									
Low	139 (40.8%)	40 (47.1%)	34 (40.0%)	35 (41.2%)	30 (34.9%)	37 (43.0%)	27 (31.8%)	29 (34.1%)	46 (54.1%)
Medium	109 (32.0%)	26 (30.6%)	25 (29.4%)	29 (34.1%)	29 (33.7%)	26 (30.2%)	24 (28.2%)	34 (40.0%)	25 (29.4%)
High	93 (27.3%)	19 (22.4%)	26 (30.6%)	21 (24.7%)	27 (31.4%)	23 (26.7%)	34 (40.0%)	22 (25.9%)	14 (16.5%)
Cigarette smoking status, *n* (%)									
Never	129 (37.8%)	17 (20.0%)	40 (47.1%)	44 (51.8%)	28 (32.6%)	35 (40.7%)	30 (35.3%)	29 (34.1%)	35 (41.2%)
Former	170 (49.9%)	50 (58.8%)	30 (35.3%)	37 (43.5%)	53 (61.6%)	34 (39.5%)	46 (54.1%)	46 (54.1%)	44 (51.8%)
Current	42 (12.3%)	18 (21.2%)	15 (17.6%)	4 (4.7%)	5 (5.8%)	17 (19.8%)	9 (10.6%)	10 (11.8%)	6 (7.1%)
Comorbidities, *n* (%)									
0	51 (15.0%)	7 (8.2%)	15 (17.6%)	16 (18.8%)	13 (15.1%)	8 (9.3%)	7 (8.2%)	18 (21.2%)	18 (21.2%)
1	80 (23.5%)	19 (22.4%)	22 (25.9%)	20 (23.5%)	19 (22.1%)	23 (26.7%)	23 (27.1%)	17 (20.0%)	17 (20.0%)
≥ 2	210 (61.6%)	59 (69.4%)	48 (56.5%)	49 (57.6%)	54 (62.8%)	55 (64.0%)	55 (64.7%)	50 (58.8%)	50 (58.8%)
Tumor stage, *n* (%)									
I	220 (64.5%)	49 (57.6%)	58 (68.2%)	62 (72.9%)	51 (59.3%)	57 (66.3%)	51 (60.0%)	53 (62.4%)	59 (69.4%)
II	53 (15.5%)	17 (20.0%)	11 (12.9%)	7 (8.2%)	18 (20.9%)	9 (10.5%)	17 (20.0%)	14 (16.5%)	13 (15.3%)
III	68 (19.9%)	19 (22.4%)	16 (18.8%)	16 (18.8%)	17 (19.8%)	20 (23.3%)	17 (20.0%)	18 (21.2%)	13 (15.3%)
Tumor grade, *n* (%)									
1	44 (14.5%)	13 (16.5%)	9 (12.0%)	14 (17.9%)	8 (11.1%)	12 (15.0%)	8 (10.1%)	14 (19.2%)	10 (13.9%)
2	174 (57.2%)	46 (58.2%)	48 (64.0%)	44 (56.4%)	36 (50.0%)	48 (60.0%)	43 (54.4%)	36 (49.3%)	47 (65.3%)
3	66 (21.7%)	16 (20.3%)	14 (18.7%)	16 (20.5%)	20 (27.8%)	18 (22.5%)	22 (27.8%)	18 (24.7%)	8 (11.1%)
4	20 (6.6%)	4 (5.1%)	4 (5.3%)	4 (5.1%)	8 (11.1%)	2 (2.5%)	6 (7.6%)	5 (6.8%)	7 (9.7%)
Missing	37	6	10	7	14	6	6	12	13
Type of surgery, *n* (%)									
Radical nephrectomy	197 (57.8%)	58 (68.2%)	46 (54.1%)	42 (49.4%)	51 (59.3%)	45 (52.3%)	58 (68.2%)	51 (60.0%)	43 (50.6%)
Partial nephrectomy	140 (41.1%)	24 (28.2%)	39 (45.9%)	43 (50.6%)	34 (39.5%)	38 (44.2%)	26 (30.6%)	34 (40.0%)	42 (49.4%)
Other	4 (1.2%)	3 (2.6%)	0 (0.0%)	0 (0.0%)	1 (2.6%)	3 (2.6%)	1 (1.2%)	0 (0.0%)	0 (0.0%)
Grade ≥ 2 postoperative complication, *n* (%)	64 (18.8%)	23 (27.1%)	13 (15.3%)	14 (16.5%)	14 (16.3%)	17 (19.8%)	16 (18.8%)	19 (22.4%)	12 (14.1%)
MVPA (h/week), mean (SD)	4.8 (2.9)	1.8 (0.7)	3.5 (0.5)	5.1 (0.6)	9.0 (2.1)	3.4 (2.2)	4.7 (2.7)	5.0 (2.6)	6.2 (3.3)
Sedentary time (h/day), mean (SD)	10.1 (1.7)	10.9 (1.8)	10.5 (1.6)	9.8 (1.4)	9.3 (1.7)	12.3 (0.8)	10.7 (0.3)	9.6 (0.4)	7.9 (0.9)
Mobility limitation, *n* (%)									
No	252 (73.9%)	45 (52.9%)	62 (72.9%)	73 (85.9%)	72 (83.7%)	61 (70.9%)	64 (75.3%)	60 (70.6%)	67 (78.8%)
Yes	89 (25.5%)	40 (47.1%)	23 (27.1%)	12 (14.1%)	14 (16.3%)	25 (29.1%)	21 (24.7%)	25 (29.4%)	18 (21.2%)

^a^Including 1 patient with underweight (BMI < 18.5 kg/m^2^)

**Fig. 1:**
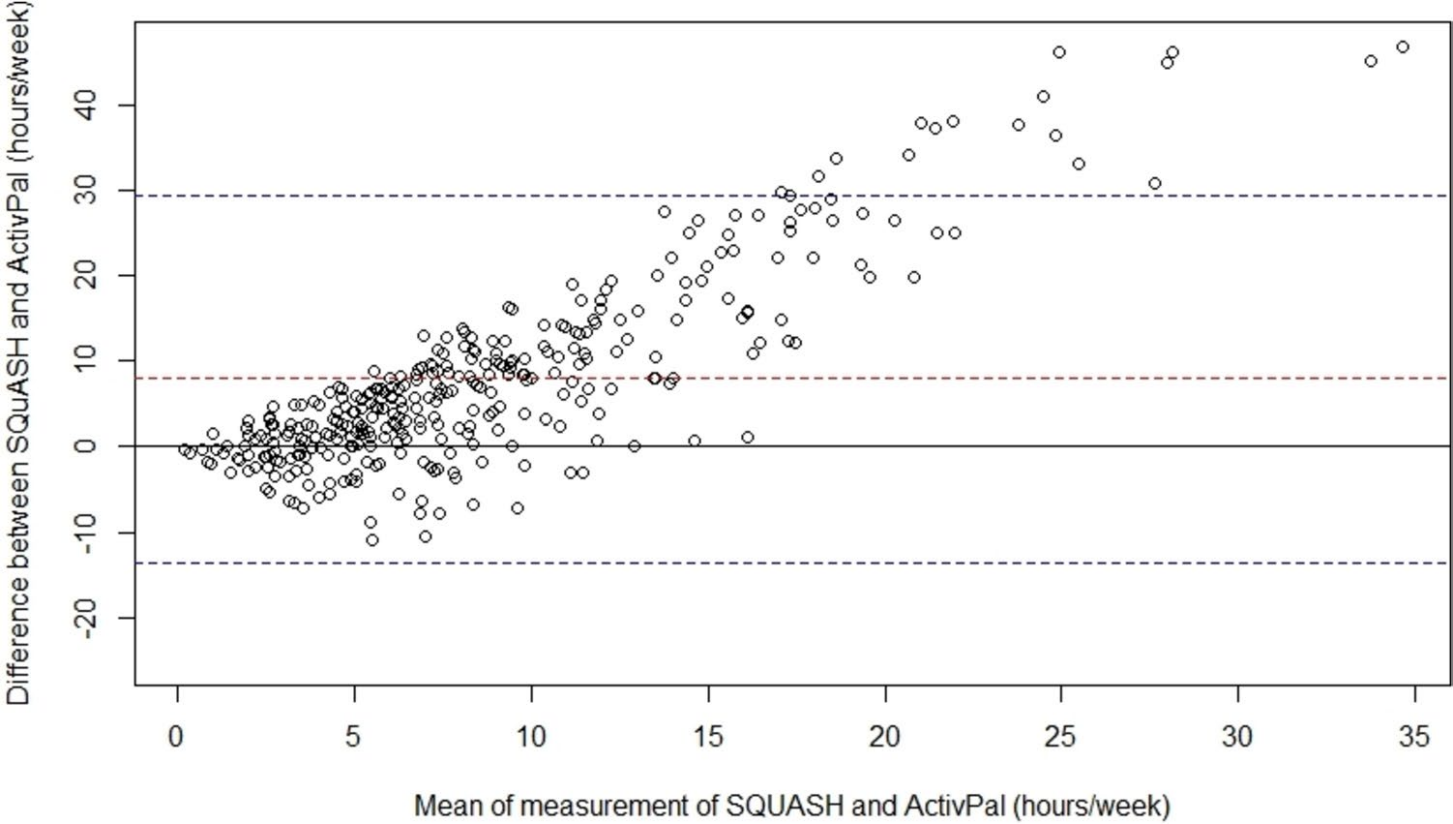
Bland–Altman plot for the agreement of moderate-to-vigorous physical activity of SQUASH and ActivPal. The dashed red line represents the mean bias (7.91, SD = 10.97 h/week), the dashed blue lines the upper (29.41 h/week) and lower (− 13.59 h/week) 95% limit of agreement, and the solid line perfect agreement

Descriptive statistics for the device-measured MVPA and sedentary time data and HRQoL measures are shown in Table 2. The device and questionnaire data were collected on average 101 (SD 29) and 99 (SD 30) days after surgery, respectively. Mean wearing time was 6 (SD 0.6) days, and mean time spent on MVPA and sedentary time was 4.8 (SD 2.9) h/week and 10.1 (SD 1.7) h/day, respectively. The mean self-reported MVPA was 12.7 (SD 11.1) h/week. Adherence to the WCRF/AICR recommendations was 77% for device-measured MVPA, while it was 88% for self-reported MVPA. The convergent validity of self-reported MVPA and device-measured MVPA was weak (Spearman’s Rho = 0.19). Bland–Altman analysis showed that self-reported MVPA assessed by SQUASH was systematically higher when compared to device-measured MVPA assessed by ActivPAL with a mean difference of 7.91 ± 10.97 h/week, and the 95% limits of agreement ranging between − 13.59 and 29.41 h/week (Fig. 1). Agreement on classifying participants as meeting the WCRF/AICR recommendations on MVPA using these methods (kappa = 0.01) was poor.

**Table 2:** Descriptive statistics for ActivPal-measured physical activity, sedentary time, and health-related quality of life of patients with localized RCC

Variable	Mean (SD)	Median (IQR)
Physical activity and sedentary behavior		
Time between treatment and ActivPal measurement, days	101 (29)	94 (86, 105)
ActivPal valid days	6.0 (0.6)	6 (6, 6)
ActivPal valid week days	4.4 (0.6)	4 (4,5)
ActivPal valid weekend days	1.6 (0.5)	2 (1, 2)
Moderate-to-vigorous physical activity, h/week	4.8 (2.9)	4.2 (2.7, 6.4)
Sedentary time, h/day	10.1 (1.7)	10.2 (8.8, 11.4)
Health-related quality of life and fatigue		
Time between treatment and questionnaire, days	99 (30)	91 (84, 102)
Global quality of life	76.5 (17.3)	79.2 (66.7, 83.3)
Physical functioning	87.1 (14.6)	93.3 (80.0, 100)
Role functioning	80.6 (26.3)	100 (66.7, 100)
Emotional functioning	84.0 (18.9)	75 (66.7, 91.7)
Cognitive functioning	85.6 (20.1)	100 (83.3, 100)
Social functioning	83.7 (22.1)	100 (66.7, 100)
QLQ C-30 summary score	86.2 (12.0)	89.7 (79.4, 95.5)
Fatigue	26.5 (22.3)	22.2 (11.1, 33.3)

### MVPA and HRQoL

The highest (≥ 6.7 h/week) versus the lowest quartile (≤ 2.7 h/week) of device-measured MVPA was significantly associated with better HRQoL scores for global health status (*β*, 10.2; 95% CI, 5.1, 15.3), physical (*β*, 7.7; 95% CI, 3.8, 11.6), role (*β*, 12.4; 95% CI, 4.7, 20.2), and social (*β*, 7.3; 95% CI, 0.2, 14.4) functioning, and the summary score (*β*, 4.6; 95% CI, 1.1, 8.1), and with lower fatigue (*β*, − 11.2; 95% CI, − 18.1, − 4.2) (Table 3). These differences were of small clinical relevance, except for the difference in global health status which was of medium clinical relevance [27]. Most second and third quartiles of MVPA were also associated with better scores on the same HRQoL domains, but effect estimates were smaller. Individuals adhering to the WCRF/AICR recommendations of 150 min/week MVPA compared to those not adhering had significantly better HRQoL scores for the same outcomes, except for social functioning (Table 3).

**Table 3:** The association of device-measured and self-reported moderate-to-vigorous physical activity (MVPA) and device-measured sedentary time with health-related quality of life (HRQoL)

	Global health status	Physical functioning	Role functioning	Emotional functioning	Cognitive functioning	Social functioning	Summary score	Fatigue
	*β* (95% CI)	*β* (95% CI)	*β* (95% CI)	*β* (95% CI)	*β* (95% CI)	*β* (95% CI)	*β* (95% CI)	*β* (95% CI)
**Device-measured MVPA in quartiles (h/week)**^**a,b**^								
0–2.7	Ref	Ref	Ref	Ref	Ref	Ref	Ref	Ref
2.8–4.2	7.7 (2.9, 12.5)*	7.6 (3.9, 11.2)*	8.2 (1.0, 15.5)*	0.2 (− 5.6, 6.0)	− 0.8 (− 7.2, 5.7)	2.6 (− 4.1, 9.3)	2.8 (− 0.5, 6.1)	− 5.9 (− 12.4, 0.6)
4.2–6.7	5.9 (0.8, 11.0)*	5.8 (1.9, 9.6)*	9.3 (1.6, 17.0)*	0.2 (− 5.9, 6.3)	− 0.6 (− 7.5, 6.2)	1.9 (− 5.1, 8.9)	3.7 (0.2, 7.1)*	− 7.8 (− 14.6, − 0.9)*
≥ 6.7	10.2 (5.1, 15.3)*	7.7 (3.8, 11.6)*	12.4 (4.7, 20.2)*	1.7 (− 4.5, 7.9)	0.6 (− 6.3, 7.5)	7.3 (0.2, 14.4)*	4.6 (1.1, 8.1)*	− 11.2 (− 18.1, − 4.2)*
*p*-trend	0.001	0.001	0.003	0.60	0.83	0.06	0.01	0.002
**Device-measured MVPA continuous (h/week)**^**a,b**^								
1 unit increase	1.1 (0.4, 1.7)*	0.7 (0.2, 1.2)*	1.3 (0.3, 2.2)*	0.2 (− 0.5, 1.0)	− 0.1 (− 0.9, 0.8)	0.8 (− 0.1, 1.6)	0.5 (0.0, 0.9)*	− 1.3 (− 2.2, − 0.5)*
**Device-measured MVPA in categories of WCRF/AICR recommendations (min/week)**^**a,b,c**^								
< 150	Ref	Ref	Ref	Ref	Ref	Ref	Ref	Ref
≥ 150	7.9 (3.6, 12.3)*	8.0 (4.7 (11.3)*	11.0 (4.5, 17.5)*	0.5 (− 4.8, 5.7)	− 0.0 (− 5.8, 5.8)	3.9 (− 2.1, 9.9)	3.6 (0.6, 6.5)*	− 7.5 (− 13.4, − 1.6)*
**Self-reported MVPA in quartiles (h/week)**^**a**^								
0–4.8	Ref	Ref	Ref	Ref	Ref	Ref	Ref	Ref
5.0–9.5	2.5 (− 2.1, 7.1)	5.5 (2.0, 9.0)*	7.8 (0.9, 14.7)*	0.4 (− 5.1, 5.8)	0.4 (− 5.7, 6.5)	3.3 (− 3.1, 9.6)	1.3 (− 1.8, 4.5)	− 5.8 (− 12.0, 0.3)
9.5–16.8	6.6 (1.8, 11.3)*	7.6 (4.0, 11.2)*	9.0 (1.9, 16.1)*	0.5 (− 5.1, 6.2)	0.7 (− 5.6, 7.0)	3.6 (− 2.9, 10.1)	2.5 (− 0.7, 5.7)	− 6.2 (− 12.6, 0.1)
≥ 17.0	5.2 (0.5, 10.0)*	8.4 (4.8, 11.9)*	9.4 (2.4, 16.5)*	− 1.8 (− 7.4, 3.8)	1.6 (− 4.7, 7.8)	4.9 (− 1.5, 11.4)	2.3 (− 0.9, 5.5)	− 5.8 (− 12.2, 0.5)
*p*-trend	0.01	< 0.001	0.01	0.55	0.61	0.15	0.12	0.09
**Self-reported MVPA continuous (h/week)**^**a**^								
1 unit increase	0.1 (− 0.0, 0.3)	0.2 (0.1, 0.3)*	0.3 (0.1, 0.5)*	− 0.1 (− 0.3, 0.1)	0.0 (− 0.2, 0.2)	0.1 (− 0.1, 0.3)	0.1 (− 0.0, 0.2)	− 0.1 (− 0.3, 0.1)
**Self-reported MVPA in categories of WCRF/AICR recommendations (min/week)**^**a,d**^								
< 150	Ref	Ref	Ref	Ref	Ref	Ref	Ref	Ref
≥ 150	2.8 (− 2.4, 8.0)	4.4 (0.4, 8.3)*	4.9 (− 2.9, 12.6)	− 2.3 (− 8.4, 3.8)	− 1.2 (− 8.0, 5.6)	− 1.4 (− 8.5, 5.6)	0.2 (− 3.3, 3.7)	− 2.3 (− 9.3, 4.6)
**Device-measured sedentary time in quartiles (h/day)**^**a,e**^								
≥ 11.4	Ref	Ref	Ref	Ref	Ref	Ref	Ref	Ref
10.2–11.4	− 1.4 (− 6.1, 3.3)	0.2 (− 3.4, 3.7)	− 6.1 (− 13.1, 0.9)	− 4.0 (− 9.6, 1.6)	− 5.7 (− 11.9, 0.5)	− 5.8 (− 12.3, 0.6)	− 1.9 (− 5.1, 1.3)	4.4 (− 1.9, 10.6)
8.8–10.2	0.8 (− 3.9, 5.6)	3.8 (0.2, 7.4)*	0.7 (− 6.4, 7.8)	− 1.7 (− 7.4, 3.9)	− 3.0 (− 9.3, 3.3)	− 0.1 (− 6.5, 6.4)	1.4 (− 1.9, 4.6)	− 0.5 (− 6.8, 5.8)
0–8.8	1.3 (− 3.7, 6.3)	4.6 (0.8, 8.5)*	− 2.3 (− 9.8, 5.2)	− 4.9 (− 10.9, 1.1)	− 3.8 (− 10.5, 2.9)	− 2.2 (− 9.0, 4.7)	− 0.9 (− 4.3, 2.6)	1.3 (− 5.4, 8.0)
*p*-trend	0.46	0.005	1.00	0.20	0.40	0.94	0.89	0.93
**Device-measured sedentary time continuous (h/day)**^**a,e**^								
1 unit decrease	0.5 (− 0.6, 1.5)	1.2 (0.4, 2.0)*	0.5 (− 1.1, 2.1)	0.7 (− 1.9, 0.6)	− 0.3 (− 1.7, 1.0)	0.2 (− 1.2, 1.7)	0.2 (− 0.5, 0.9)	− 0.2 (− 1.6, 1.2)

^*^p < 0.05

^a^Model is adjusted for age (years), sex (male, female), BMI (kg/m^2^), comorbidities (0, 1, ≥ 2), tumor stage (I, II, III), smoking status (never, former, current), education level (low, medium, high), and mobility limitation (yes, no)

^b^Model for device-measured MVPA is also adjusted for sedentary time (h/day)

^c^0–150 (*n* = 77), ≥ 150 (*n* = 264)

^d^0–150 (*n* = 40); ≥ 150 (*n* = 300)

^e^Model for sedentary time is also adjusted for MVPA (h/week)

For the highest (≥ 17.0 h/week) versus the lowest quartile (≤ 2.7 h/week) of self-reported MVPA, similar associations were found for global health status (*β*, 5.2; 95% CI, 0.5, 10.0), physical (*β*, 8.4; 95% CI, 4.8, 11.9), and role (*β* 9.4; 95% CI, 2.4, 16.5) functioning. Associations for social functioning, the summary score, and fatigue were in the same direction but not statistically significant (Table 3).

### Sedentary time and HRQoL

The lowest (≤ 8.8 h/day; *β*, 4.6; 95% CI, 0.8, 8.5) and second (8.8–10.2 h/day; *β*, 3.8; 95% CI, 0.2, 7.4) versus the highest quartile (≥ 11.5 h/day) of sedentary time was associated with better physical functioning (Table 3). Differences were below the 5-point threshold for a small clinically relevant difference. No significant associations between sedentary time and the other HRQoL domains were observed.

## Discussion

In this cross-sectional study, we investigated the association between device-measured MVPA and sedentary time and self-reported MVPA with HRQoL at 3 months after surgery in patients with localized RCC. Higher device-measured MVPA and meeting the WCRF/AICR physical activity recommendations were significantly associated with better global quality of life, physical, role, and social functioning, and the summary score, and with less fatigue. Associations for self-reported MVPA were in the same direction but weaker. Lower sedentary time was significantly associated with better physical functioning but not with other domains of HRQoL.

We found that higher device-measured MVPA was associated with higher scores on several HRQoL domains including less fatigue. Patients with localized RCC are an understudied population with respect to MVPA and sedentary time in relation to HRQoL including fatigue. Although primary treatment mainly involves surgery, patients’ HRQoL can still be affected by side effects [4]. Indeed, HRQoL levels in our study were lower and fatigue levels were higher compared to published values of a Dutch normative population [28]. The results of studies conducted on other cancer survivors were mixed. For example, some studies in patients with breast, prostate, or lung cancer showed no clear associations of device-measured MVPA with HRQoL or fatigue [29–31] while other studies in patients with breast and colorectal cancer showed similar associations as in our study [32–35]. These mixed results may be explained by differences in patient characteristics, cancer type, stage, and (timing after) treatment.

We showed that lower device-measured sedentary time was only associated with higher physical functioning and not with other HRQoL outcomes or fatigue. A systematic review on sedentary time and health outcomes among cancer survivors including ten studies with data on device-measured sedentary time and HRQoL or fatigue did not support a clear association either [7]. Only four cross-sectional studies among patients with breast [29], colorectal [36], lung cancer [31], or mixed cancers [37] found an association of sedentary time with HRQoL [31], physical functioning [31, 36], or fatigue [29, 31, 36]. These primarily null observations have been suggested to be due to the high HRQoL and the low fatigue levels in the included study populations [7]. However, three more recent studies among breast [34, 35] and colorectal survivors [38] did observe an association of higher sedentary time with lower HRQoL and/or higher fatigue outcomes.

The convergent validity between device-measured and self-reported MVPA in our study was low and the bias was high, as also described in previous studies comparing device-measured and self-reported physical activity in adults [11, 13, 39]. This can be explained by the fact that both measures capture different aspects of physical activity as well as by recall and social desirability bias inherent to self-report [11, 13]. Despite this low correlation, we found that associations with HRQoL were in the same direction but slightly stronger for device-measured compared to self-reported MVPA, underscoring the robustness of our findings. Interestingly, a study among 1348 survivors of breast, prostate, and colorectal cancer also found similar associations for device-measured and self-reported MVPA with fatigue while only self-reported MVPA was found to be associated with HRQoL [40].

Our results for self-reported MVPA are consistent with findings from one previous cross-sectional study among 463 Canadian patients with RCC reporting an association between self-reported physical activity and HRQoL at 6 years after diagnosis [10]. In contrast to our study, only 26% of patients reported to be moderately to vigorously active for at least 150 min/week. Numerous cross-sectional studies in other cancer populations also reported associations between higher self-reported MVPA with better HRQoL outcomes [41–43].

According to the guidelines for the interpretation of the EORTC QLQ-C30 effect estimates [27], the mean differences for HRQoL outcomes between the highest vs. lowest quartiles of device-measured and self-reported MVPA were of small clinical relevance, which is considered subtle but nevertheless clinically relevant [27]. A medium clinically relevant difference, considered likely to be clinically relevant [27], was only found for device-measured MVPA and global quality of life. These results are in line with an observational study among patients with breast cancer showing small effect sizes for device-measured MVPA and HRQoL and fatigue 3 months after surgery before the start of adjuvant therapy [35].

Although our results indicate that more than three quarters of our study population adhered to the WCRF/AICR recommendations for cancer prevention to engage in at least 150 min/week of MVPA, there is still room for improvement. It is currently unclear whether the relation of MVPA with HRQoL and fatigue is causal as patients may also have less MVPA because of a lower HRQoL or more fatigue (i.e. reverse causality). Thus, based on our results we can only suggest that increasing MVPA might be an intervention target to improve HRQoL and reduce fatigue in patients with localized RCC after surgery. Future longitudinal studies and intervention studies should be conducted to obtain better insight into the causality of our observed associations. Until more evidence becomes available, following the WCRF/AICR recommendations to engage in at least 150 min/week of MVPA and to minimize the sedentary time is advisable, also because of the potential beneficial effects on other health outcomes.

This study had several strengths. To the best of our knowledge, this is the first study to investigate the association between device-measured MVPA and sedentary time in patients with localized RCC. The validated activPAL3 monitors, which are a highly accurate and valid tool for measuring sedentary, standing, and stepping time [16], were worn for 24 h/day for an average of 6 days, providing an accurate reflection of MVPA and sedentary time during the post-surgery recovery phase. Also, extensive data on potential confounders, such as BMI, smoking, and mobility limitation, were collected and taken into account in the analyses.

There are also limitations to consider. First, our cross-sectional study design limits the ability to draw conclusions regarding causality, and we need to be aware that findings may also be explained by reverse causality. Second, the use of activPAL3 accelerometers to calculate MVPA does not account for physical activity other than stepping movements. As a result, not all movements (e.g., upper body movements; push and pull exercises which are frequently performed during fitness) were captured in the total time spent in MVPA. Future studies might benefit from combining thigh-worn accelerometers with chest-mounted tri-axial accelerometers measuring free weight exercises [44]. Third, the activPAL3 monitor does not provide information on the context within which physical activity and sedentary behavior are occurring. Different domains of physical activity (e.g. commuting, recreational, sports) [45] but possibly also sedentary behavior (e.g. office work, watching television) may be differently related to HRQoL, and accelerometers cannot take this context into account. Fourth, the response rate in our study was 44% and we cannot rule out that healthier patients or those with a healthier lifestyle were more likely to participate, also because activity levels in our population were relatively high. This may affect the generalizability of our findings. However, except for being slightly more likely to be female, participants of this study were comparable to invited non-participants with respect to age, tumor stage, tumor grade, morphology, and type of treatment while other (lifestyle-related) characteristics of non-participants were not available [15]. Lastly, residual confounding by unmeasured factors cannot be excluded.

In conclusion, both higher device-measured and self-reported MVPA were independently associated with significantly better HRQoL outcomes including less fatigue among patients with localized RCC, with differences being of small clinical relevance. Lower sedentary time was only associated with better physical functioning. Our findings add to the current evidence that advice on increasing MVPA may also be effective for patients with localized RCC to improve HRQoL including fatigue outcomes while decreasing sedentary time seems less relevant. Future longitudinal studies and intervention studies should provide insight into whether the observed associations are causal. This information can contribute to the development of targeted MVPA guidelines and interventions to improve health outcomes in this patient group.

## Data Availability

The data that support the findings of this study are available upon request from the corresponding author.
